# An action framework for the participatory assessment of nature-based solutions in cities

**DOI:** 10.1007/s13280-022-01772-6

**Published:** 2022-08-06

**Authors:** Alexander P. N. van der Jagt, Arjen Buijs, Cynnamon Dobbs, Martina van Lierop, Stephan Pauleit, Thomas B. Randrup, Tom Wild

**Affiliations:** 1 Forest and Nature Conservation Policy Group, Department of Environmental Sciences, Wageningen University & Research, Droevendaalsesteeg 3, 6708 PB Wageningen, The Netherlands; 2 Wageningen Environmental Research, Droevendaalsesteeg 3, 6708 PB Wageningen, The Netherlands; 3 Center for Modeling and Monitoring Ecosystems, School of Forestry, Universidad Mayor, Jose Toribio Medina 29, Santiago, Chile; 4 Chair for Strategic Landscape Planning and Management, School of Life Sciences Weihenstephan, Technical University of Munich, Emil-Ramann-Str. 6, 85354 Freising, Germany; 5 Department of Landscape Architecture, Planning and Management, Swedish University of Agricultural Sciences, Lomma, Box 190, 234 22 Lomma, Sweden; 6 Department of Landscape Architecture, University of Sheffield, Floor 13, Arts Tower, Western Bank, Sheffield, S10 2TN UK

**Keywords:** Co-production, Ecosystem services, Environmental justice, Nature-based thinking, Participatory monitoring, Urban nature-based solutions

## Abstract

Impact assessment is a key step in mainstreaming urban nature-based solutions (NBS). Yet, it remains unclear if and how assessment frameworks influence urban planning, design and management. We contend that the potential of current NBS assessment frameworks is not fully exploited due to: (1) limited contextualisation of monitoring and assessment to place-specific contexts and (2) the depoliticisation of co-production. To address this, we present a practical five-step action framework to guide inclusive participation across different stages of monitoring and assessment of urban NBS, including indicator selection. Unlike previous approaches, applying selection criteria at the level of individual indicators, we also use criteria at the aggregate level of the indicator set. We conclude that participatory assessment contributes to mainstreaming urban NBS for sustainable and just cities, provided data is contextualised to local decision-making contexts and the process is designed to amplify marginalised voices.

**Supplementary Information:**

The online version contains supplementary material available at 10.1007/s13280-022-01772-6.

## Introduction

Nature-based solutions (NBS) represent innovative and cost-effective interventions, including sustainable urban drainage systems and communal gardens, tapping into the potential of nature to help create more resilient ecosystems and societies (European Commission, EC 2021). NBS can be particularly promising for cities given their vulnerabilities to climate change and environmental degradation, and the associated detriment in air quality, thermal comfort, drinking water supply, environmental justice and social cohesion (e.g. Depietri and McPhearson 2017; Hobbie and Grimm 2020; Xie and Bulkeley 2020). Although on the rise, NBS are not yet mainstream in urban development (Dorst et al. 2021; Frantzeskaki and McPhearson 2021).

There is a growing understanding of the need to develop more participatory approaches to mainstream NBS in urban planning, involving co-design, co-production and co-management. This reflects a broader trend in sustainability and other societal domains towards co-production of solutions by researchers and societal stakeholders (Chambers et al. 2021; Kleinhans et al. 2022). Whereas for some time co-production was used by states as a method for improving government effectiveness, current understandings require co-produced solutions to respond to the challenges prioritised by citizens (Watson 2014). The complexity and uncertainty of urban development processes and challenges, such as climate change, requires a transdisciplinary approach in which knowledge from different citizens and communities, practices and scientific disciplines is brought together (Buijs et al. 2016; Frantzeskaki et al. 2019; Norström et al. 2020). Dealing with these issues is now “a shared responsibility of state, market and civil society” (Lange et al. 2013, p. 404).

Co-design, co-production and co-management need to be tailored to place-specific contexts to be relevant, effective and successful (Norström et al. 2020) and, in the case of urban greening, improve human–nature relationships (Frantzeskaki et al. 2019). In our culturally diverse cities, this means considering the social–cultural values and needs from a heterogeneous group of stakeholders (Buijs et al. 2016). Yet, co-production is not without bias and can be marred by unbalanced power relations and social capital (Norström et al. 2020). For participatory approaches to contribute robustly to urban NBS for sustainable and just cities, more empowering forms of co-production are needed (Wamsler et al. 2020). This implies that local government should go beyond tokenistic consultation or placation by delegating power and work in partnership with societal actors, starting from shared goal formulation to the co-production of data and planning support systems (Pan et al. 2022).

While collaborative urban NBS planning, design and implementation is increasingly considered (Janse and Konijnendijk 2007; Buijs et al. 2016; Fors et al. 2021), we still see limited evidence of co-produced monitoring and assessment approaches for urban NBS. For example, IUCN assessment of NBS in accordance with their Global Standard can only be done by centrally trained and accredited professionals (IUCN n.d.), which is likely cost prohibitive to marginalised regions and local grassroots initiatives. This is problematic given that diverse knowledge user participation is crucial to the uptake of environmental assessment in practice and its impact on real-world decision making (Saarikoski et al. 2018; McQuatters-Gollop et al. 2019; Rogers et al. 2020), including for assessment frameworks on NBS, biodiversity and ecosystem services (Giordano et al. 2020; Stevance et al. 2020; Coletta et al. 2021).

In line with the understanding that improved data, assessment and metrics could contribute to urban NBS mainstreaming (van der Jagt et al. 2020; Tozer et al. 2022),  considerable research funding was made available to support the development of NBS data and metrics. Since 2015, the EU alone has funded over 20 research projects developing assessment approaches for (urban) NBS and related concepts (Dumitru and Wendling 2021). The majority of these draw on the EKLIPSE impact evaluation framework by Raymond et al. (2017) as a mechanism for organising indicators corresponding to various NBS co-benefits by policy sectors and societal challenge areas (e.g. climate resilience, public health and wellbeing and economic opportunities and green jobs). Alternative assessment frameworks often have a narrower scope on e.g. climate vulnerabilities or climate resilience contributions of NBS (Calliari et al. 2019; Beceiro et al. 2020; Shah et al. 2020) or to green infrastructure and other specific types of NBS (Artmann and Sartison 2018; Lee and Oh 2019). Frameworks have also been developed for monetising NBS co-benefits (Shiao et al. 2020), and measuring performance against core NBS design principles (IUCN 2020). Despite ongoing investment in urban NBS assessment frameworks, available examples still lack comprehensive guidance regarding the various stages of participatory assessment.

Recently, the EC synthesised the insights accrued from these studies into a single compendium—the practitioner handbook on evaluating the impact of NBS (Dumitru and Wendling 2021). In line with the argument for participatory assessment, it calls for co-creating a theory of change to guide indicator selection, along with a shared monitoring and evaluation strategy. Unlike many of the assessment frameworks underpinning the handbook, it also recommends a transdisciplinary approach, social engagement through citizen science and a policy-relevant approach drawing on available, accessible and reusable data. Therefore, it is more ambitious on participatory assessment than most EU-funded alternatives. However, we believe the EC practitioner handbook still lacks a convincing rationale for why participatory assessment is vital for increasing societal impact. Their practical guidance is mainly focused on the data collection stage of participatory assessment, while other stages in the learning cycle of (adaptive) NBS co-production remain neglected. Therefore, the objectives of this Perspective article are to (1) critically appraise the current standard of co-production in urban NBS monitoring and assessment, (2) discuss the transformative potential of infusing monitoring and assessment with local perspectives and (3) provide a practical way forward with an action framework for participatory assessment to be used in conjunction with current NBS assessment frameworks.

## Nature-based thinking as a theoretical lens

**Fig. 1:**
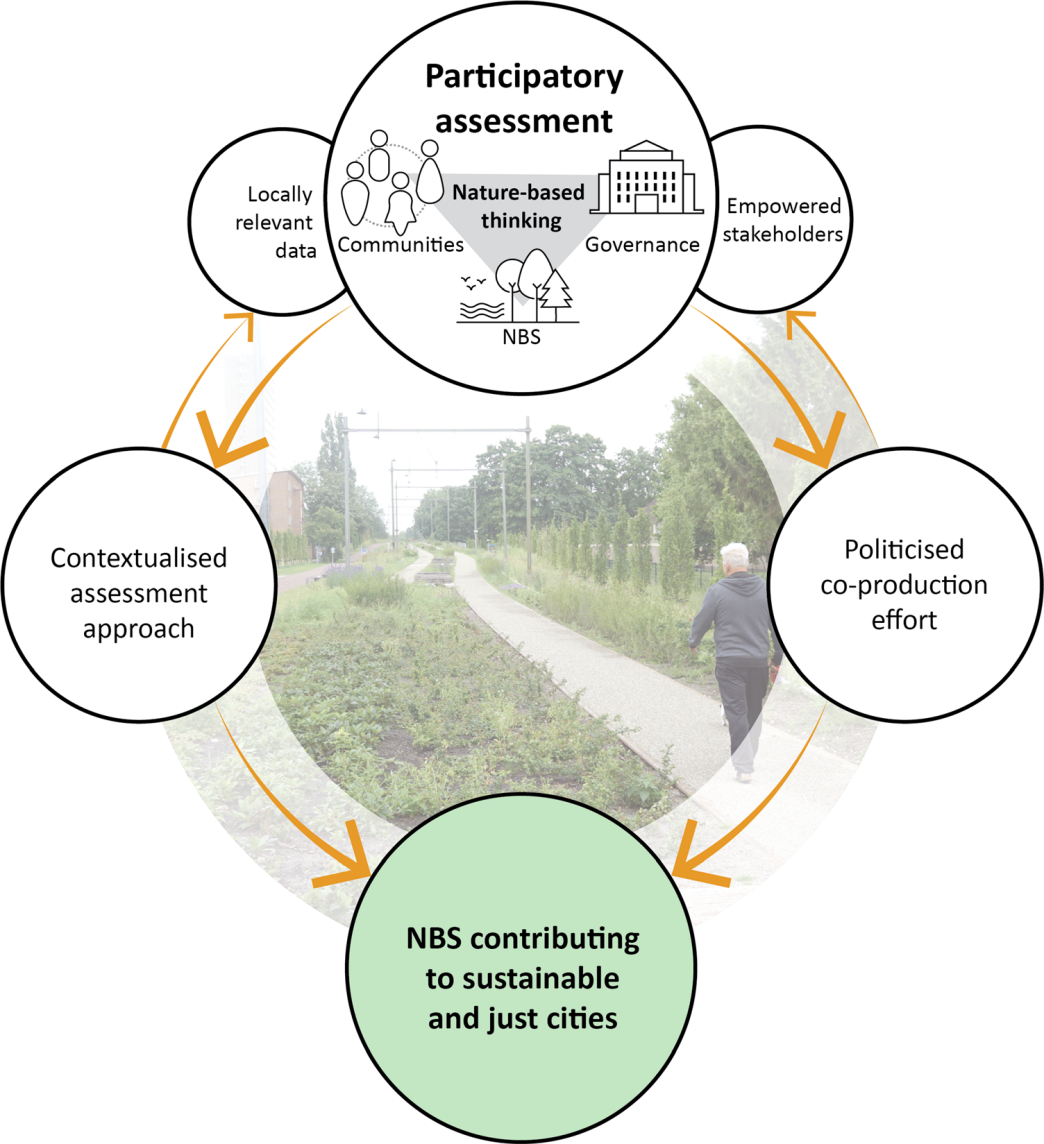
Nature-based thinking as a theoretical lens for participatory assessment of urban NBS. The photo was taken by Arjen Buijs

As shown in Fig. 1, we draw on the concept of nature-based thinking (NBT) to argue how extensive and inclusive participation in NBS assessment might contribute to urban NBS mainstreaming. NBT can be understood as a relational mindset considering culturally diverse communities, institutional governance and thriving NBS as interlinked, rather than as isolated phenomena (Randrup et al. 2020). Here, we consider the development of NBT as a prerequisite for the contribution of assessment to NBS mainstreaming because with “recognising that humans are an indivisible part of nature, the current Anthropocene also implies a responsibility towards the re-generation of nature, especially in cities” (Randrup et al. 2020, p. 6). Building on previous frameworks for integrated sustainability assessment by e.g. Weaver and Rotmans (2006) and Hurley et al., (2010), the development of NBT at personal, communal and institutional levels relies on widespread stakeholder participation in environmental stewardship and a collaborative and reflexive approach to the development of knowledge on NBS. When NBS assessment is responsive to each of the communities–environment, communities–institutions and institutions–environment nexuses of NBT, it becomes more *contextualised*, thereupon generating more useful data.

Strengthening interconnections between culturally diverse communities, institutions and NBS through NBT also requires an effort to *politicise* assessment, which we understand as challenging the asymmetries in power influencing whose knowledge is prioritised in decisions and solutions (Turnhout et al. 2020). This is key to participation’s transformative potential and associated processes of environmental commitment and value shifts (Lawrence 2006). Therefore, participatory assessment should engage local stakeholder groups with underrepresented and potentially unconventional knowledge (needs) along with powerful policy makers and practitioners in reflexive arrangements (van der Jagt et al. 2021). While contextualised assessment responds to NBT, politicised assessment seeks to strengthen NBT across communities and institutions. Consequently, a positive feedback loop between NBT and contextualised and politicised assessment can be established (Fig. 1).

## The limited contribution of existing assessment frameworks to sustainable and just cities

A lack of contextualised data and the depoliticisation of co-produced data are key to understanding why the potential of assessment for more sustainable and just cities is not fully exploited. We support our argument, where relevant, with a small number of semi-structured interviews (*N* = 4) with coordinators or lead researchers of the top five of relevant EU-funded NBS assessment frameworks (Table 1; Method provided in the Supplementary Information). These interviews addressed the uptake of the NBS assessment framework by cities—where and how—during and beyond the project duration, the level and type of participation afforded and the usability of the framework across different contexts.

**Table 1:** The integration of participatory assessment principles in five recent and influential EU-funded urban NBS assessment frameworks

Assessment framework	Aim	Scope for participatory assessment?	Source
Connecting Nature Impact Assessment Guidebook	To guide the implementation of a robust assessment approach for NBS	It includes a call for stakeholder engagement and provides guidance on co-developing a theory of change to align indicators with urban priorities	Dumitru and Lourido (2022)
EKLIPSE impact evaluation framework for nature-based solutions	To support the generation of common evidence and a knowledge base for NBS, specifically for assessing climate resilience benefits at different geographic scales	Not explicitly discussed beyond the identification of participatory mapping as a potential method	Raymond et al. (2017)
European Commission Handbook for NBS assessment	To develop a common framework for integrated NBS assessment for all H2020 NBS projects, which can be used as a reference for common indicators by NBS projects and EU policy	Scope for participatory data collection is described for each indicator and the value of citizen science is emphasised. It also includes a section on adapting indicators to decision-making contexts using stakeholder engagement and co-developing a theory of change	Dumitru and Wendling (2021)
NATURVATION Urban Nature Navigator	To help stakeholders understand their sustainability priorities and evaluate the potential of different types of urban NBS in meeting these priorities	It provides generic guidance on participatory and deliberative methods to guide assessment, but this information is not provided at the level of individual indicators	Dammers et al. (2019)
UnaLab NBS Performance and Impact Monitoring Protocols	To provide practitioners with metrics for assessing NBS benefits along with guidance for monitoring these	No	Wendling et al. (2019)

### The lack of relevant, contextualised data

**Fig. 2:**
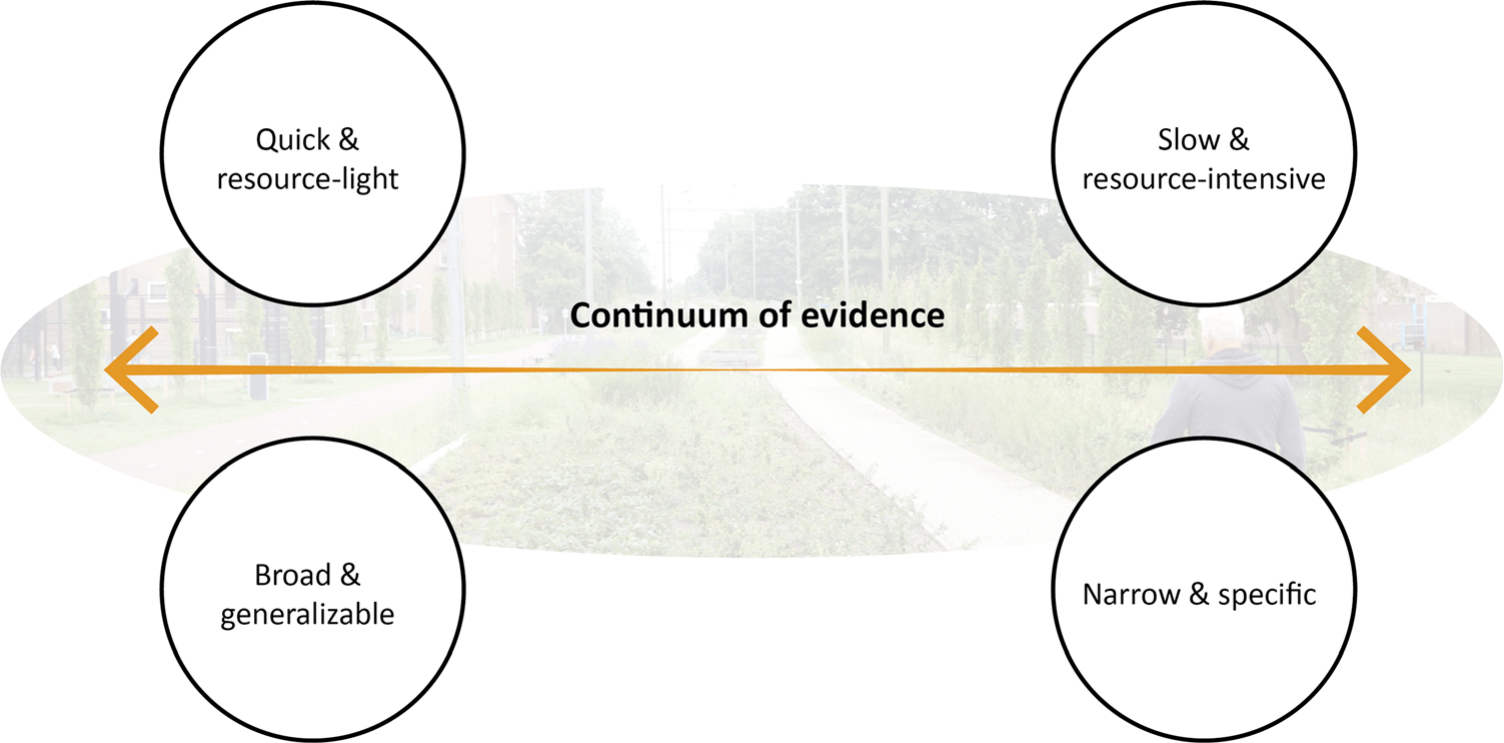
The continuum of evidence by DeMeo et al. (2015) (authors’ interpretation)

Assessment framework developers often assume that real-world decision making is informed by scientific facts and modernist worldviews. In reality, however, urban municipalities often have a rather pragmatic and opportunistic approach to urban green space monitoring and environmental management (Carmen et al. 2020). The abstract, complex and detailed monitoring regimes advocated by developers of scientific assessment frameworks tend to require considerable funding, knowledge and time to implement and maintain. Therefore, they are frequently deemed unrealistic, complicated, cost-prohibitive and poorly aligned with policy agendas and planning regulations (DeMeo et al. 2015; Evans and Guariguata 2016; van Oudenhoven et al. 2018b; Carmen et al. 2020; Rogers et al. 2020). Limited relevance of evaluated impacts to policy and everyday practice can result in stakeholders losing interest to participate (Stevance et al. 2020): “*No matter how much interdisciplinary scientists think they are over-simplifying biophysical or socio-economic processes, decision-makers typically ask for simpler, easy-to-use and understandable decision support tools that can be readily incorporated into science-policy processes*” (Ruckelshaus et al. 2015, p. 17). The *continuum of evidence* (Fig. 2) aptly illustrates this tension between scientists—typically striving for complex technical information specific to a particular NBS, scale or benefit and potential end-users (e.g. urban planners) looking for a resource-light and broadly applicable approach they can manage in-house. For example, a scientist might seek to measure carbon sequestration in soils and vegetation in urban forests, whereas a planner would like to be able to paint a broader picture of the various benefits provided by urban forests relevant to meeting environmental, social and economic policy goals using a few quick and easy-to-measure metrics. A successful collaboration requires them to agree on an optimal position on this continuum (DeMeo et al. 2015).

A related issue is that science-based assessment framework developers are often oblivious to how their indicators align with knowledge, working routines, actor network dynamics, policy frameworks and other institutional structures (Rydin et al. 2003). “*I think municipalities have very strong cultures of how things are done and it's very difficult to change those cultures if you're not part of it, or not aware of it*” (Int. #1). Therefore, rather than changing cultures, one should aim to align with these while acknowledging that everything that is adding to, rather than easing, work routines is unlikely to stick beyond a project’s life span. Hence, researchers are encouraged to propose indicators optimally suited to the socio-political and socio-cultural contexts in which practitioners operate. This should not be interpreted as excluding possibilities to challenge entrenched institutional structures and routines, as reflected in the parallel need to politicise the co-production effort.

### The depoliticisation of co-production

Turnhout et al. (2020) highlight three ways in which co-production can be politicised to contribute to more inclusive and just societies: (1) scientific knowledge is not to be prioritised over other ways of knowing; (2) the co-production process should expose fundamental differences in vulnerability, risk and resources between groups; and (3) the process should engage with higher level political processes relevant to the project.

To ensure that *scientific knowledge is not prioritised over other ways of knowing* there is a need to leverage local and, where relevant, indigenous knowledge in assessment approaches. This was aptly expressed by one of the interviewed NBS assessment framework developers: “*There was quite a bit of imposition of knowledge on us in a way* [when designing the assessment framework], *and I would want to think more about not only how to engage with indigenous and local knowledge outside of the team, but also how to take a more inclusive approach to team development, so that everyone feels like they have a common vision and mission in terms of the assessment*” (Int. #4)*.*

This could be addressed by identifying local data sources and ways of measuring or understanding phenomena: “*The chapter in our Handbook may be a bit Eurocentric because we've talked a lot about sources of European data. So maybe that's less applicable to South America or outside Europe in general. But I'm sure that analogous data sources can be identified*” (Int. #3). Citizen science offers potential advantages as one way of integrating local knowledge and experiences. Despite increasing interest in co-producing assessment approaches with stakeholders, the interviews revealed that citizen contributions are often less actively pursued. This is problematic because despite its lower level of scientific rigour, community engagement through e.g. citizen science has the potential to support environmental awareness and citizen empowerment as well as long-term continuity of assessment regardless of political dynamics (Savan et al. 2003; Bonney et al. 2009; Dickinson et al. 2012). Citizen science can be supported with the use of e-tools such as public participation geographic information systems (PPGIS; e.g. Rall et al. 2019), which has been widely used in NBS planning. Contextualised knowledge can also be accessed via volunteered geographic information (VGI; Gulsrud et al. 2018; Steen Møller et al. 2019; Wild et al. 2019), e.g. regarding the ways people with different ethnicities, ages, gender identities and socio-economic status experience and interact with the environment, including the topic of justice in urban development. A challenge is, however, to develop a user-friendly tool drawing on indicators perceived as useful by citizens, urban practitioners and scientists alike (Pocock et al. 2018), which does justice to the emotional investment by citizens in environmental care and conservation. Citizen data should, therefore, never be treated as a commodity to be traded with third parties (Lawrence and Turnhout 2010), while assurances need to be provided around data accessibility and transparency regarding who is using it for which purposes.

For a participatory assessment approach to *expose fundamental differences in vulnerability, risk and resources between groups*, there is an urgent need to monitor environmental justice as a core societal challenge area across all NBS projects and regardless of who is involved. The distinction between core and supplementary indicators is in line with good practice adopted in previous projects: “*The core indicators reflect as far as possible [the] indicators that would be applicable across very different NBS projects, at different scales as well to some extent. […] I guess the core indicators reflect what would be the minimum you would need to measure*” (Int. #2). Supplementary indicators apply only to particular NBS projects, depending on context-specific challenges and priorities (Dumitru and Lourido 2022).

This data should not only be monitored but also analysed and disseminated comprehensively through *engagement with higher level political processes relevant to the project*, including institutions and potential investors in NBS. A key benefit of doing so is that institutions might be deterred from selectively using only some indicators, while ignoring others, to serve a narrow pre-defined interest rather than the public interest—i.e. policy-based evidence as opposed to evidence-based policy (Sharman and Holmes 2010). Consequently, engaging with powerful agents is key to NBS becoming a tool for community building rather than community displacement (Toxopeus et al. 2020; Kotsila et al. 2021; van der Jagt et al. 2021). Developing an assessment framework for urban sustainability action should, therefore, be less about inventing and fine-tuning indicators, and more about monitoring and improving their role in urban governance (Rydin et al. 2003).

## Key features of participatory assessment

**Fig. 3:**
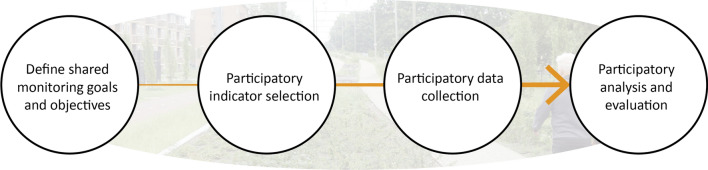
Stages in participatory monitoring and assessment

Participatory monitoring and assessment have been conceptualised in various ways. The World Bank, widely cited on this topic (e.g. Matsiliza 2012), take a particular focus on sharing decision-making powers with stakeholders in the stages of data collection and analysis, which also provides scope for challenging dominant practices (Atkins and Wildau 2008). Others consider a broader range of activities relevant to assessment, including project co-design, participatory data collection and analysis (Evans et al. 2018). To ensure appropriate contextualization, we extend this common conceptualization with the stage of participatory indicator selection (also see Bautista et al. 2017 and Morris and Lawrence 2010). Accordingly, we define participatory monitoring and assessment as an iterative science-policy interface engaging urban stakeholders, including marginalised voices, in (1) *defining shared monitoring goals and objectives*, (2) *participatory indicator selection*, (3) *participatory data collection* and (4) *participatory data analysis and evaluation* (Fig. 3).

The participatory development of assessment approaches provides a variety of benefits. First, it supports evidence-based planning resulting from improved indicator uptake (Mickwitz and Melanen 2009). Second, it helps to generate new and relevant data and ideas, which benefits organisational learning capacity and institutional effectiveness (Atkins and Wildau 2008; Fernandez-Gimenez et al. 2008; Reed 2008; Tarrasón et al. 2016). Third, engaging civil society in monitoring and assessment could help to leverage sense of place, social cohesion, biocultural diversity and social learning (Fernandez-Gimenez et al. 2008; Krasny et al. 2014; Buizer et al. 2016; Sinclair and Diduck 2017; Uchiyama and Kohsaka 2019). Fourth, it increases stakeholder commitment to monitoring and a sense of shared ownership of this process (Morris and Lawrence 2010; DeMeo et al. 2015; Evans and Guariguata 2016; Viani et al. 2017). Fifth, it empowers marginalised stakeholders as a result of improved skills and knowledge, growth of social capital within the community and a more relational approach to the stewardship of local environments considering communities’ habits, traditions and worldviews (Lawrence 2006; Constantino et al. 2012; Bautista et al. 2017). Sixth, it can lead to stronger public support for urban NBS as the local knowledge and values influencing co-produced assessment indicators might eventually help to improve NBS designs and management procedures (Neumann and Hack 2022).

To structure and simplify the co-selection of indicators, there is a need for adopting principles, or criteria guiding participatory assessment. We draw inspiration from sustainable development literature engaging with the theory–practice gap, which shows that participatory assessment not only requires indicators that are scientifically *credible*, but also *legitimate* (i.e. inclusive, unbiased and fair) and *salient* (i.e. relevant to knowledge users) (Cash et al. 2003). A more recent synthesis paper on ecosystem services indicator development highlighted *feasibility* as a fourth main indicator selection criterion (van Oudenhoven et al. 2018a). Out of these criteria, feasibility and salience were found to be central to decisions urban municipalities make about which indicators to use for urban green space monitoring (Carmen et al. 2020). Feasibility has, however, been operationalised in different ways. Whereas in aforementioned studies it was operationalised as availability of data, time, finance and expertise, broader definitions also include aspects of legitimacy (i.e. social feasibility), along with political and legal dimensions (Patterson et al. 2021), influencing e.g. the degree to which urban planning approaches are evidence based. We recommend adopting this broader conceptualisation of feasibility, taking into account the socio-political context of a city, to better contextualise urban NBS assessment.

In sum, to be successful, participatory assessment coordinators need to navigate the complexities of urban decision making, including resource constraints, competing political agendas and entrenched institutions, norms and practices (i.e. *contextualisation*). At the same time, they should also take into account the knowledge needs of diverse urban societies and stakeholders. When designed with this principle in mind, participatory assessment creates an opening for improved *politicisation* of co-produced data, contributing to the empowerment of marginalised groups and the mainstreaming of a relational NBT mindset among communities and institutions.

## An action framework for the participatory assessment of urban NBS

Academics play a crucial role in participatory assessment by improving awareness about the co-benefits of NBS, linking these to sustainability challenges, strengthening the argument and developing and advancing the state-of-the-art indicators to assess these. However, they often face challenges around the contextualisation and politicisation of their assessment approaches. To overcome these, we synergise lessons observed to various extents in previous assessment frameworks for urban NBS (Int. #2; Int. #3; Dammers et al. 2019; Wendling et al. 2019; Dumitru and Wendling 2021; Dumitru and Lourido 2022), which are presented in Steps 1–4 below. Going beyond prevailing benchmarks for participatory assessment, we add a new step (Step 5) on evaluating the locally adopted assessment approach as a whole, rather than the individual indicators, on criteria for politicised and contextualised assessment. Together, these actions represent the action framework for participatory assessment of urban NBS. Implementing the action framework will strengthen the positive feedback loop between NBT and contextualised and politicised assessment (Fig. 1). We discuss each of the five steps below.

**Step 1**. The first step in participatory monitoring and assessment is to perform stakeholder mapping and to decide on who to engage at which stage of the participatory assessment process. Relevant stakeholder groups include public institutions, academia and research organisations, civil society organisations, community representatives and the private sector, with ideally a balanced number of participants from each of these groups. Supportive methods and approaches are available enabling a legitimate approach (Reed 2008; van der Jagt et al. 2019).

After relevant stakeholders have been identified, they should be engaged—using a focus group or workshop—in **the definition of shared monitoring goals and objectives** corresponding with the desired effects of the collaborative project and particular projects (e.g. Dumitru and Wendling 2021). Separate objectives should be formulated at the stakeholder or network level (e.g. capacity building) and at level of individual NBS projects (e.g. improved social cohesion; DeMeo et al. 2015; Evans and Guariguata 2016). These latter objectives can be formulated for different scales (e.g. macro, meso, micro), depending on project scope. Following the example of Dumitru and Wendling (2021), selected objectives should be mapped onto societal challenge areas (e.g. climate resilience, biodiversity enhancement or social justice) to guide the identification of potentially relevant indicator portfolio sources.

Selected objectives should be co-developed with various stakeholders, including local government and civil society, to correspond with their knowledge needs: “*[…] there was a lot of trial and error, but what was most helpful in the end was actually turning the process around from what is state-of-the-art science to, let's start from your priorities and then we will support you […]. And once they started getting into it, they became very much in use and they're actually very enthusiastic about the indicators*” (Int. #2)*.* Adaptive co-management, an iterative approach to reconsidering goals and objectives over time, needs to be adopted for responding to evolving understandings of what it means to accomplish these (Huitema et al. 2009; Pahl-Wostl 2017). Step 1, therefore, does not have a clear end-point.

**Step 2**. The next step in setting up a participatory monitoring process is to collate a credible indicator portfolio aligned with locally relevant societal challenge areas and desired scale(s) of measurement. This should come along with the provision of basic descriptive information on required data, measurement procedure, scale of measurement, measurement unit and scope for citizen science (for an excellent example, see Dumitru and Wendling 2021). We recommend to draw indicators from an up-to-date scientific source framework—such as the EC practitioner handbook—with indicators clustered based on societal challenge areas (e.g. climate resilience). This needs to include indicators that are relevant for the urban context, desired scale(s) of measurement and the assessment of process or governance dynamics. If relevant, this main source for indicators could be supplemented with indicator frameworks specifically tailored to monitoring a focal issue or certain geographic context (e.g. Forest Stewardship Council criteria for sustainable urban forestry). In addition, scientists and local stakeholders should be given the opportunity to suggest complementary indicators based on their understanding of relevant challenges and available data, and experience of monitoring these.

**Step 3**. Following this, the assessment coordinator makes a first (pre)selection of indicators from the portfolio based on the co-defined monitoring goals and objectives (Neugarten et al. 2018; Dumitru and Wendling 2021). Selection criteria are applied to ensure that each indicator is: (1) aligned with a locally relevant societal challenge and measurement scale(s), (2) relevant to the urban context, (3) suitable for monitoring NBS impacts within the timeframe of the measurement period and (4) requiring no specialist expertise going beyond the (short- and long term) organisational resource availability for monitoring and assessment. Data requirements for indicators should also be considered. For example, an indicator drawing on an existing European dataset might not be fit for purpose in Latin America. We recommend to cap the number of pre-selected indicators in such a way that stakeholders are provided with options to choose, yet can still manage to deliberate each indicator within the timeframe of a single workshop (see Step 4).

**Step 4**. To gain feedback on the pre-selected indicators used for the final selection of indicators, a stakeholder indicator appraisal workshop is conducted involving public institutions, civil society, academia and the private sector. This should be a light-touch and undemanding event by managing indicator numbers and minimising detailed technical information: “*The ones getting involved in the project are not always data experts or impact assessment experts of any kind*” (Int. #2). This workshop should be carefully designed to give everyone a fair chance to contribute. First, it needs to be ensured all stakeholders involved approve of the project objectives. Second, indicators for different projects are deliberated. This is best done in separate (break-out) sessions for each project to prevent stakeholder confusion about the project or scale to which objectives apply. Third, participants deliberate and score salience (“How relevant is this indicator for evaluating if the objective(s) is met?”). This exercise is ideally repeated for other criteria such as feasibility: “*Go for the ideal and keep that somewhere [in your consciousness] because even if you are only going to measure a few indicators right now […], you might see that actually in the next project or in the next policy funding cycle, you can include [those other ones too]*” (Int. #2). As a minimum, we recommend deliberating and scoring indicators on both salience and feasibility. Existing assessment frameworks for urban NBS also call for organising stakeholder workshops to identify indicators for monitoring progress against achieving project objectives, based on developing a theory of change with desired short, medium and long-term changes (Int. #2; Int. #3; Dumitru and Wendling 2021; Dumitru and Lourido 2022). We drew inspiration from NATURVATIONs Urban Nature Navigator to include questions about perceived relevance and feasibility (referred to as legitimacy in the original framework) of indicators (Dammers et al. 2019).

**Fig. 4:**
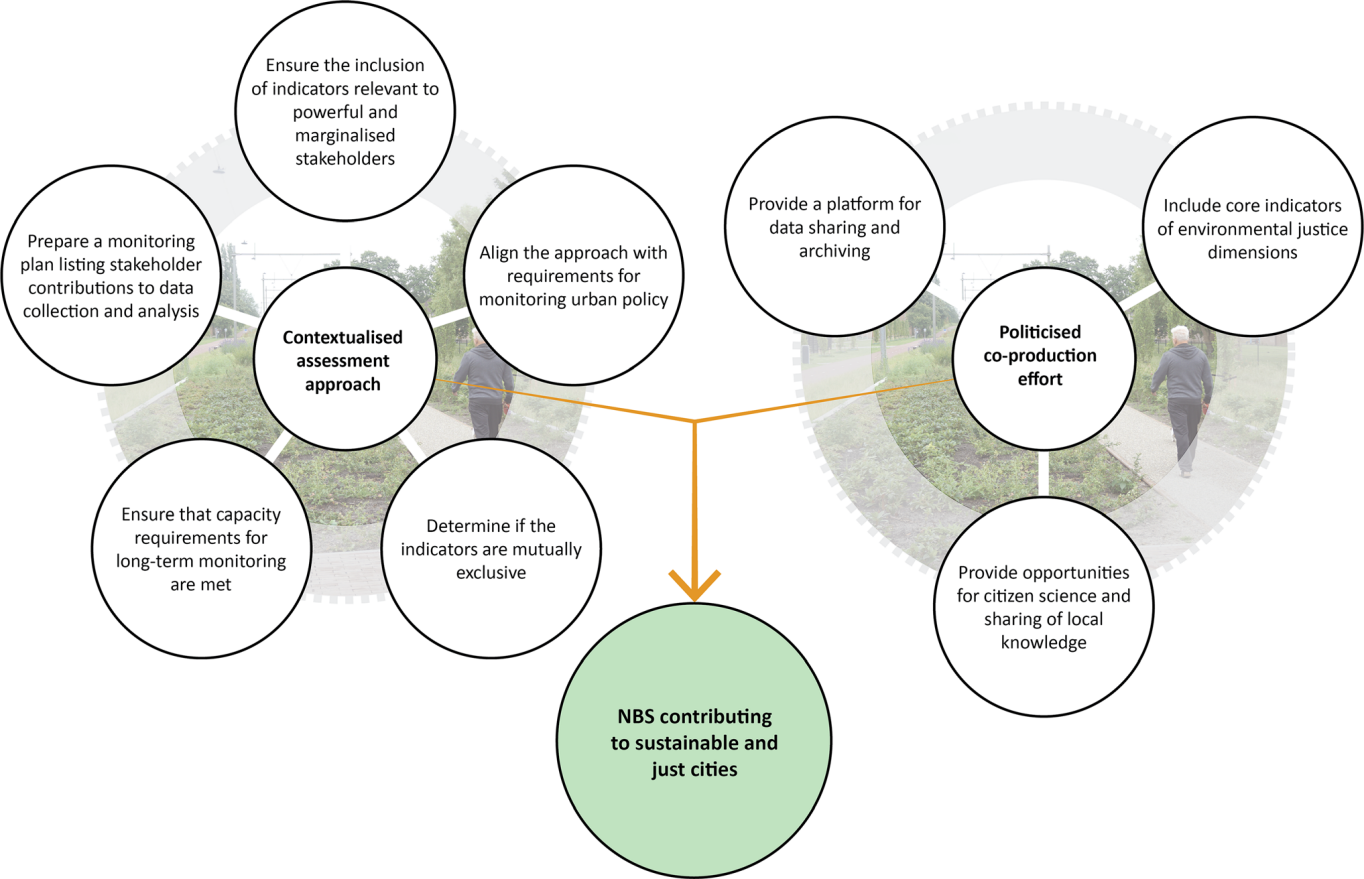
Criteria for politicised and contextualised assessment applied at the level of the full set of indicators (Step 5 of the action framework)

**Step 5**. The final step, going beyond prevailing practices in any of the studied assessment frameworks for urban NBS, is to apply criteria for politicised and contextualised assessment. These are not introduced at the level of the individual indicator (Steps 3 and 4) but at the aggregate level of the (preliminary) assessment approach, comprising the full set of indicators and measurement methods derived from the indicator appraisal workshop. Figure 4 provides an overview of relevant criteria and how these relate to the contextualisation and politicisation of monitoring and assessment.

The selected assessment approach needs to reflect the interests of a broad and representative group of stakeholders to ensure its legitimacy. While van Oudenhoven et al. (2018a) applied legitimacy as a criterion to select individual indicators (similar to salience and feasibility in Step 4), we contend it should instead be evaluated for the assessment approach as a whole. This is because not every indicator has to be legitimate to a broad range of stakeholders, especially if it would generate knowledge currently underrepresented in urban decision making, e.g. because it responds to the interests of marginalised groups. This is notwithstanding the importance for the assessment approach to generate data relevant to urban policy goals and targets across the social, environmental and economic domains. Moreover, it should be verified if sufficient institutional capacity is available, not just for measuring and analysing individual indicators, but also for implementing the assessment approach as a whole. And not just during the funded research period, but also beyond. Indicators generating overlapping or similar data should be avoided when resources are stretched. Once the assessment approach is finalised, a monitoring plan should be developed specifying who will coordinate the assessment of particular indicators and at which intervals (Evans and Guariguata 2016).

At a minimum, the following actions are required to achieve a politicised assessment approach. To enable participation of marginalised groups and sharing of local knowledge, it is crucial to ascertain that at least some of the selected indicators are appropriate for citizen science. Moreover, core indicators around environmental justice should always be included, preferably measuring this in a comprehensive way drawing on multiple justice dimensions. To empower marginalised stakeholders in using data for political lobbying and activism, data transparency and accessibility is key (Gulsrud et al. 2018). Therefore, we encourage the development of a digital infrastructure for data storage and analysis, using e.g. mobile apps or an online inventory (Evans and Guariguata 2016). Training in particular measurement and analytical techniques by researchers or other project partners needs to be arranged, where relevant.

## Conclusion

Building upon and extending current approaches to co-production, we argued that participatory monitoring and assessment is key to support improved mainstreaming of urban NBS for sustainable and just cities. A review of the most important EU-funded NBS assessment frameworks revealed that existing approaches are often insufficiently sensitive to specific political and socio-cultural contexts, which limits their long-term uptake and impact on decision making. To better align assessment with institutional structures, policy targets and the knowledge needs of local stakeholders, all stages of monitoring and assessment should be made more participatory, including the indicator selection. Moreover, there is a requirement for politicising assessment by monitoring, analysing and disseminating environmental justice impacts for all NBS projects. Doing so across communities and institutions will likely amplify marginalised voices in urban planning, design and management, unfolding the transformative potential of NBS assessment. Together, processes of contextualising and politicising assessment improve the potential for urban NBS mainstreaming through the delivery of more usable data along with empowered local stakeholders and communities able to carry forward the assessment approach beyond the duration of a fixed-term collaborative research project.

We advocated that successful participatory assessment relies on relational NBT, where the aim is not simply to produce solutions, but to initiate long-term processes enabling data to become an instrument for strengthening the connections between institutions and nature, as well as between institutions and the ways diverse stakeholder communities relate to nature. Improving current guidance in this area, we offered five practical steps towards supporting participatory, co-produced, assessment—from the joint definition of monitoring goals and objectives to applying criteria for politicised and contextualised assessment.

This action framework for participatory assessment supports cities in generating a co-produced assessment approach for urban NBS, which is contextualised to their local decision-making context. Moreover, this framework allows consideration of diverse knowledge systems, increases awareness of environmental justice and provides scope for stakeholder empowerment. Our action framework was designed to be simple, pragmatic and accessible to researchers and municipalities with varying budgets for stakeholder engagement. Although it does not require an expensive training programme to master, there remains a need for devoting considerable resources to stakeholder engagement and social learning activities. However, these investments are likely outweighed by the improved co-benefits of NBS developed in this way.

Future research should explore how participatory assessment could go beyond merely engaging urban stakeholders by actively supporting the empowerment of marginalised citizens, which would likely require trained facilitators and the uptake of various co-production techniques enabling their voices to be heard (Watson 2014). This would pave the way for leveraging the maximum synergistic potential from combining state-of-the-art scientific indicators with a broad spectrum of local knowledge, key to creating sustainable and just cities.

## Supplementary Information

Below is the link to the electronic supplementary material.

Supplementary file1 (PDF 694 kb)
