# Supplementary material for: An action framework for the participatory assessment of nature-based solutions in cities
Source: Ambio. 2022 Aug 6;52(1):54–67. doi: 10.1007/s13280-022-01772-6 (PMC9666616; doi:10.1007/s13280-022-01772-6)
Supplement: Supplementary file 1 — Supplementary file1 (PDF 694 kb) [file 13280_2022_1772_MOESM1_ESM.pdf]

Ambio

Supplementary Information

This supplementary information has not been peer reviewed.

Title: **An action framework for the participatory assessment of nature-based solutions in cities**

Methods statement

We interviewed four developers of EU-funded NBS assessment frameworks. In all, 13 local experts were interviewed, mostly from the municipality (see Table S1). Relevant interview contacts were identified based on a review of the portfolio of NBS research and innovation projects funded through the European Commission's Horizon 2020 programme. The identified NBS assessment frameworks identified in this way were ranked based on relevance for this research based on the following criteria relevant to the CONEXUS research project:

- Indicators with a scientific basis;
- a specific focus on NBS assessment;
- covers multiple sustainability challenges;
- NBS benefits are explicitly linked to sustainability challenges;
- relevant to the urban context;
- distinguishes indicators for micro, meso and macro scales;
- includes process or governance indicators; and
- describes scope for participatory assessment.

The top five assessment frameworks, shown in Table S1, were selected. Relevant documentation on these frameworks was retrieved through an online search and four assessment approach developers were identified based on this. Two of the identified developers had been involved in the development of two different assessment frameworks and were interviewed on both frameworks. The interviews were conducted between March and May 2021.

Table S1. Interview data.

**Interview #**	**Assessment framework**	**Role**	**Organisation**
1	NATURVATION Urban Nature Navigator	Co-coordinator of assessment framework development	Netherlands Environmental Assessment Agency, The Netherlands
2	• Connecting Nature Impact Assessment Guidebook<br>• European Commission Handbook for NBS assessment	Coordinator (both frameworks)	University of A Coruña, Spain
3	• UnaLab NBS Performance and Impact Monitoring Protocols<br>• European Commission Handbook for NBS assessment	Coordinator (both frameworks)	VTT Technical Research Centre of Finland, Finland
4	EKLIPSE impact evaluation framework for nature-based solutions	Lead coordinator	University of Helsinki, Finland

Using a semi-structured interviewing approach, we covered four broad themes:

1. The uptake of the NBS assessment framework and indicators during the collaborative project, for which purposes, and at which locations and scales
2. The uptake of the NBS assessment framework and indicators beyond the project, including the transferability to other cities
3. Level of participation in monitoring and assessment afforded by the assessment framework
4. The role of socio-cultural, socio-economic and political context in assessment, and opportunities for improved contextualisation

The duration of the interviews was approximately one hour. All of the interviews took place online using Teams or Zoom as communication platform. All interviews were conducted in the English language, audio-recorded and transcribed. The transcripts were thematically coded using the software package ATLAS.ti based on the main themes highlighted above, with inductive coding applied to highlight particular subthemes.

Ethical permission for this research was obtained by the Social Sciences Ethics Committee of Wageningen University. All interview participants were sent an information sheet with background information on this research ahead of their participation. Participants provided informed consent ahead of participating in the interview.

Semi-structured interview guide

The following overview provides a list of questions and topics that were covered during the interviews:

1. Could you introduce yourself and your role in the development of the assessment framework?
2. What are the main differences between this and other assessment frameworks that you are aware of?
3. Have you engaged with urban stakeholders during the development of your assessment framework? In which way(s) (e.g., scoping, developing, testing)?
4. Did urban stakeholders influence the selection of indicators? In which way?
5. To what extent does the framework allow for participation in data collection and analysis?
6. Did urban stakeholders apply the assessment framework during the project? At which scale? Did they use all indicators? If using only a selection, why?
7. Did you get any feedback on the applicability of the indicators by urban stakeholders? What was this about? Has this been used to improve the framework?
8. Are you aware of urban stakeholders continuing to use indicators from the assessment framework beyond the research project's duration? Which ones? Why (not)?
9. Does your assessment framework provide practical guidance to practitioners on how to select indicators most suited to their context? If yes, could you summarize the main points in this guidance?
10. With the knowledge you have now, do you perceive any areas for improvement in the assessment framework?
